# Significance of the gut tract in the therapeutic mechanisms of polydopamine for acute cerebral infarction: neuro-immune interaction through the gut-brain axis

**DOI:** 10.3389/fcimb.2024.1413018

**Published:** 2025-03-04

**Authors:** Feng-Hua Xu, Xiao Sun, Jun Zhu, Ling-Yang Kong, Yuan Chang, Ning Li, Wen-Xiang Hui, Cong-Peng Zhang, Yi-Ming Cheng, Wen-Xin Han, Zhi-Min Tian, Yan-Ning Qiao, Dong-feng Chen, Lei Liu, Da-Yun Feng, Jing Han

**Affiliations:** ^1^ Key Laboratory of Modern Teaching Technology, Shaanxi Normal University, Xi’an, China; ^2^ State Key Laboratory of Holistic Integrative Management of Gastrointestinal Cancers and Xijing Hospital of Digestive Diseases, Air Force Medical University, Xi’an, China; ^3^ Department of Gastroenterology, Chongqing Key Laboratory of Digestive Malignancies, Daping Hospital, Army Medical University (Third Military Medical University), Chongqing, China; ^4^ Department of Neurosurgery and Institute for Functional Brain Disorders, Tangdu Hospital, Fourth Military Medical University, Xi’an, China; ^5^ College of life sciences, Shaanxi Normal University, Xi’an, China; ^6^ Key Laboratory of Special Functional and Smart Polymer Materials of Ministry of Industry and Information Technology, School of Chemistry and Chemical Engineering, Northwestern Polytechnical University, Xi’an, China

**Keywords:** acute cerebral infarction, brain-gut axis, polydopamine, reactive oxygen species, inflammatory responses

## Abstract

**Background:**

Recent research has made significant progress in elucidating gastrointestinal complications following acute cerebral infarction (ACI), which includes disorders in intestinal motility and dysbiosis of the gut microbiota. Nevertheless, the role of the gut (which is acknowledged as being the largest immune organ) in the immunoreactive effects of polydopamine nanoparticles (PDA) on acute ischemic stroke remains inadequately understood. In addition to its function in nutrient absorption, the gut acts as a protective barrier against microbes. Systemic immune responses, which are triggered by the disruption of gut barrier integrity, are considered as one of the mechanisms underlying acute ischemic stroke, with the gut-brain axis (GBA) playing a pivotal role in this process.

**Methods:**

In this study, we used a PDA intervention in an ACI model to investigate ACI-like behavior, intestinal barrier function, central and peripheral inflammation, and hippocampal neuron excitability, thus aiming to elucidate the mechanisms through which PDA improves ACI via the GBA.

**Results:**

Our findings indicated that as ACI mice experienced dysbiosis of the gut microbiota and intestinal barrier damage, the levels of proinflammatory factors in the serum and brain significantly increased. Additionally, the activation of astrocytes in the hippocampal region and neuronal apoptosis were observed in ACI mice. Importantly, our study is the first to provide evidence demonstrating that PDA effectively suppresses the neuroimmune interactions of the gut-brain axis and significantly improves intestinal epithelial barrier integrity.

**Conclusion:**

We hope that our discoveries will serve as a foundation for further explorations of the therapeutic mechanisms of PDA in ACI, particularly in elucidating the protective roles of gut microbiota and intestinal barrier function, as well as in the development of more targeted clinical interventions for ACI.

## 1 Introduction

Stroke, which is also known as a cerebrovascular accident, is a prevalent cerebrovascular disease and ranks as the second leading cause of global mortality and the third-leading cause of death and disability combined (GBD 2016 Neurology Collaborators, 2019; Feigin et al., 2022). Additionally, its incidence is steadily increasing, with over 13 million new cases reported annually. The primary therapeutic principle for Acute Cerebral Infarction (ACI) is the early restoration of blood perfusion, thus facilitating the recovery of cerebral oxygen supply. This process aims to reintroduce nutrients to ischemic brain tissue while eliminating harmful metabolic byproducts. Various pathogenic mechanisms for stroke, such as energy metabolism disturbances, excessive nitric oxide synthesis, inflammatory injury, oxidative stress, and neuronal apoptosis, have been proposed in recent years (Liu et al., 2015; Radak et al., 2017; Orellana-Urzúa et al., 2020; Jin et al., 2022; Alsbrook et al., 2023). Among these, inflammatory injury and oxidative stress have emerged as focal points of research. In response to detrimental stimuli, the balance between reactive oxygen species (ROS)-mediated antioxidant defense and oxidative damage is disrupted within the body. The accumulation of ROS within an organism or cells leads to oxidative stress, thus initiating a cascade of oxidative damage. This oxidative damage, coupled with subsequent inflammatory responses and excitotoxic cell death, not only severely hinders neuronal repair and functional recovery but also contributes to additional tissue injury and hemorrhagic transformation. Ischemia-activated microglia and astrocytes increase the secretion of proinflammatory cytokines, thus exacerbating the disruption of the blood–brain barrier (BBB), followed by the migration of circulating leukocytes to the injured site. These dysregulated neutrophils may release proinflammatory cytokines, proteases and ROS, thus perpetuating tissue damage and functional impairment. Consequently, the elimination of proinflammatory cytokines and reactive oxygen species to reduce tissue damage has emerged as a crucial therapeutic approach for stroke.

It is well known that neuro-related diseases are increasingly connected to the gastrointestinal (GI) tract. Brain injuries (especially ACI) have been identified as causes of GI disorders (Li et al., 2017; Hanscom et al., 2021; Yuan et al., 2021). The brain-gut axis serves as a major link between the nervous system and the GI system (Chang et al., 2022; Hillestad et al., 2022; Mayer et al., 2022). Previous studies have reported that ACI leads to a shift in the gut microbiota and metabolic products, thus triggering neuroinflammation and peripheral immune responses. Inflammation resulting from neuronal breakdown can induce alterations in central and peripheral immune components, which necessitates collaborative repair of ischemia-induced neural network damage by central and peripheral immune responses (Liu et al., 2019). Furthermore, research suggests that gut bacteria produce neuroactive compounds that can modulate neuronal function, thereby influencing behavior after ACI (Pluta et al., 2021). The gut microbiota is linked to poststroke depression, anxiety, psychological-psychiatric disorders, cognitive function, and systemic inflammation (Khoshnam et al., 2017; Yamashiro et al., 2017). Consequently, the relationship between the brain-gut axis and the pathogenesis of ACI has garnered significant attention, which contributes to the diagnosis, treatment, and further understanding of the mechanisms underlying ACI.

With the advancement of nanomedicine, various nanoparticles possessing anti-inflammatory and antioxidative properties have been formulated for the treatment of ACI. Among these, naturally occurring synthetic melanin nanoparticles, including polydopamine nanoparticles (PDA NPs), exhibit promise for clinical translation due to their robust antioxidative capabilities and excellent biocompatibility. Previous studies have indicated that in mouse models of acute peritonitis and acute lung injury, PDA reduces the generation of ROS, diminishes proinflammatory cytokines, alleviates neutrophil infiltration, and mitigates lung tissue damage (Zhao et al., 2018). In a mouse model of acute kidney injury, ultrasmall Mn^2+^-chelating, melanin-like nanoparticles can eliminate various toxic ROS and inhibit ROS-induced oxidative stress (Sun et al., 2019). Moreover, 1,8-dihydroxynaphthalene (PDH nanoparticles), which is a type of self-assembling melanin-like nanoparticle, not only serves as scavengers for free radicals to alleviate oxidative stress but also chelates calcium overload to suppress endoplasmic reticulum stress responses. They inhibit neutrophil infiltration and the secretion of proinflammatory cytokines (TNF-α and IL-6) (Lou et al., 2021). However, whether PDA can mitigate ACI through its anti-inflammatory and antioxidative effects remains unclear. Recently, researchers have shown that in the context of ACI, magnetic nanoparticles augment neuroprotection against hypoxic-ischemic injury in mesenchymal stem cells (Kim et al., 2020). This enhancement is achieved through the scavenging of reactive oxygen and superoxide species, deactivation of caspase-3, downregulation of the pro-apoptotic Bax protein, and upregulation of the anti-apoptotic Bcl-2 protein. Despite these promising findings, the therapeutic potential of polydopamine nanoparticles in the treatment of ACI via the gut-brain axis remains largely unexplored.

In this study, our objective was to elucidate the potential impacts of PDA on neuroimmune protection through the brain-gut axis, with a focus on its anti-inflammatory and antioxidant effects, as well as an exploration of the underlying mechanisms. Our findings indicated that post-ACI administration of PDA can significantly diminish the production of ROS and suppress the expression of proinflammatory cytokines. Moreover, it alleviates peripheral intestinal dysfunction, ameliorates dysbiosis in the gut microbiota, and consequently mitigates the brain-gut immune response associated with ACI, which are primarily accomplished through its anti-inflammatory and antioxidant properties.

## 2 Methods

### 2.1 Animals

In the experiment, we utilized male C57BL/6J mice aged 8-12 weeks, sourced from the SPF-grade animal facility at the Key Laboratory of Modern Teaching Technology, Ministry of Education, Shaanxi Normal University. All procedures were conducted in compliance with the requirements of the Chinese Animal Protection Committee and received approval from the Animal Ethics Committee of Shaanxi Normal University. Mice were randomly housed in cages of five individuals each, with separate ventilation, under a 12 hour light-dark cycle, and maintained at a temperature of 22 ± 2°C with a relative humidity of 55 ± 5%. Animals had ad libitum access to food and water. Prior to behavioral experiments, mice underwent a 3-day acclimatization period. All behavioral experiments and the collection of blood or tissue samples were conducted between 10:00 AM and 4:00 PM. The animals were randomly assigned to groups, and no predetermined sample size was applied. The mice were randomly assigned to the following groups: control, the acute cerebral infarction Animal Model (ACI) alone, and ACI plus polydopamine nanoparticles (ACI+PDA).

### 2.2 Constructing the acute cerebral infarction animal model

The infarction animal model was established according to previously published research (Engel et al., 2011). Specifically, the experimental animals were fixed on the operating table in supine position after inhalation anesthesia. Body temperature of the mice was maintained at 36.5°C ± 0.5°C during surgery with a heating plate. After the skin was disinfected, a midline neck incision is made and the soft tissues are pulled apart. The common carotid artery and vagus nerve were quickly exposed and separated, and the proximal end of the common carotid artery and the external carotid artery were connected. The internal carotid artery was threaded for use, and a small opening was cut at the upper end of the common carotid artery ligation, extending from the bifurcation of the common carotid artery. Afterwards, the origin of the middle cerebral artery was blocked by inserting a suture into 10 mm from the carotid bifurcation. After the operation, the body temperature was maintained at 37 ± 0.5°C with an irradiation lamp, and the respiration and heart rate were monitored.

### 2.3 Behavioral experiments

Prior to establishing the ACI cerebral ischemia animal model, a novel open field test was conducted on each animal to ensure consistency among experimental subjects before the commencement of the study. On the third day following the establishment of the ACI cerebral ischemia animal model, a series of behavioral assessments was performed on the ischemic animals. The modified neurological severity score (mNSS) (Chen et al., 2001; Duan et al., 2022) for mice was employed to evaluate the extent of neurological damage, encompassing aspects of motor function, sensation, and reflexes. Scores ranged from 0 (normal) to 18 (severe damage), with 1-6 indicating mild injury, 7-12 indicating moderate injury, and 13-18 indicating severe injury.

In terms of motor function, the tail suspension test was employed, involving the suspension of mice by their tails to observe the flexion of the fore and hind limbs and deviation of the head. The desktop walking test was conducted to assess the mice’s ability to walk normally. For sensory evaluation, a visual experiment involved tilting the mouse at a 45° angle with its forelimbs suspended and observing whether the forelimbs immediately grasped the desktop from a height of 10 cm. The tactile experiment consisted of lifting the mouse’s head 45° upward, with the forelimbs suspended, to observe any delayed limb reactions. In the ledge test, the mouse’s head was directed toward the edge of the table, and a gentle push from behind assessed the mouse’s ability to immediately grasp the table edge. For the balance test, a 170 cm-long, 2 cm-wide wooden rod was placed horizontally 70 cm above the ground to observe the mouse’s balance on the beam and its ability to maintain a stable posture. In terms of reflexes, mice were placed on a tabletop, and a cotton swab was used to touch the external auditory canal and cornea. A noise-producing cardboard was briskly moved near the ear to observe the mouse’s reaction. The cumulative scores from these assessments yielded the total mNSS score.

### 2.4 Synthesis of polydopamine

In this study, improvements to the previous modeling methods (Liang et al., 2023) were made, whereby 36 mg of dopamine hydrochloride was dissolved in 18 mL of water bath for 20 min. Subsequently, a rapid addition of 150 μL of a 1 mol/L sodium hydroxide aqueous solution (40 g/L = 40 mg/mL) was performed, followed by open stirring at 50°C for 5 h. The resulting solution was centrifuged at 15,000 rpm for 10 min, and the supernatant was discarded. The precipitate was then dispersed in water using ultrasonication, followed by another centrifugation at 15,000 rpm for 10 min. This process was repeated three times, resulting in the obtainment of PDA nanoparticles suspended in 4 mL of water at a concentration of 10 mg/mL. The synthesized PDA particles were thoroughly characterized for morphology, particle size, and distribution, as well as the infrared spectroscopic features, utilizing instruments such as scanning electron microscopy, transmission electron microscopy, nanoparticle size analyzer, and infrared spectrometer.

### 2.5 RNA extraction and reverse transcription-polymerase chain reaction

Total RNA was extracted from brain tissue using TRIzol reagent (15596026; Invitrogen), and the concentration of RNA was quantified by ultraviolet spectrophotometry at 260/280 nm (Nanodrop 2000, Thermo scientific, USA). For generation of cDNA, a PrimeScript™ 1st strand cDNA Synthesis Kit (TaKaRa, Japan) was used according to the manufacturer’s instructions. Subsequently, quantitative PCR (qPCR) was performed using SYBR™ Green PCR Master Mix (Q311-02, ChamQ Blue Universal SYBR qPCR Master Mix, Vazyme). The qPCR reaction was performed according to the following conditions: stage 1: predenaturation - reps 1 - 95°C 30 s; stage 2: cyclic reaction - reps 40 - 93°C 10 s and 60°C 30 s; stage 3: melt curve - reps 1 - 95°C 15 s, 60°C 60 s and 95°C 15s. PCR was performed on a Mx3000P (Agilent, USA) quantitative realtime detection system. The mRNA relative expression levels of target genes were normalized to the expression level of β-actin. The primers used were as follows:

il-1β: F-5′-gagcaccttcttttccttcatctt, R-5′-tcacacaccagcaggttatcatc;
tnf-α: F-5’-ctcttctgcctgctgcactttg, R-5’-atgggctacaggcttgtcactc;
il-10: F-5′-ccaagccttgtcggaaatga, R-5′-gctagaagcatttgcggtgg;
tgf-β: F-5′-gcctgagtggctgtcttttg, R-5′-ctgtattccgtctccttggttc;
β-actin: F-5′-tcatcactattggcaacgacg, R-5′-aacagtccgcctagaagcac.

### 2.6 Enzyme-linked immunosorbent assay

The changes of cytokines in serum were determined by ELISA. We employed a mouse ELISA kit BMS6002 (Mouse IL-1beta ELISA 96 tests, Thermo Fisher, USA); 88-7324-88 (Mouse TNF-α ELISA Ready-SET-Go); 88-7105-88 (Mouse IL-10 ELISA Ready-SET-Go, eBioscience); BMS608-4 (Mouse TGF beta-1 ELISA 96 tests, Thermo Fisher, USA)), following the protocols provided by the manufacturer as outlined in the accompanying instruction manual. Blood samples were collected via retro-orbital puncture at 4°C overnight and centrifuged at 3000 g for 20 min to generate serum samples next day. ELISA was performed according to kit protocols. A microplate reader (iMark, Bio-rad, Japan) was used to measure absorbance at 450 nm.

### 2.7 Triphenyltetrazolium chloride staining

We had improved on the previous modeling methods (Chiang et al., 2011). Mice were adequately anesthetized using 25% urethane, and their chest cavities were opened to expose the hearts. A 0.9% NaCl solution was perfused to extract the brain, and the harvested brain tissue was placed in a brain mold. The mold was then frozen at -20°C for 40 min, and mouse brain slices with a thickness of 1.5 mm were obtained using a blade within the brain mold. These brain slices were immersed in TTC staining solution at 37°C in the dark for 20 min. Subsequently, photographs were taken to observe the ischemic area in the brain tissue.

### 2.8 H&E staining

This simple dye combination (Chan, 2014) is capable of highlighting the fine structures of cells and tissues. Most cellular organelles and extracellular matrix are eosinophilic, while the nucleus, rough endoplasmic reticulum, and ribosomes are basophilic. Briefly, Harris’ hematoxylin (YuanYe Biotechnology, Shanghai, China) was used to stain 18 μm frozen sections for 5 min, which were then gently washed with water. Differentiation was carried out for 5 s with a 1% hydrochloric acid alcohol solution (1 mL), and the color of the slice was observed to change from blue to red. Then, after the sections were washed with tap water for 1.5 h, they were stained with 0.5% eosin (water-soluble, Aladdin BioChem Technology, Shanghai, China) for 2 min. After the aforementioned staining process, the sections were dehydrated with different concentrations of ethanol and cleared with 100% xylene. Cover slips were mounted over the sections before they were assessed and photographed via microscopy (Carl Zeiss, Oberkochen, Germany) under phase contrast at 10X magnification. The mounted slides were then examined and photographed using a microscope.

### 2.9 Masson staining

Fibrosis can occur in response to both physiological and pathological cues (Van De Vlekkert et al., 2020), including wound healing, tissue remodeling/repair and inflammation. Chronic fibrosis can lead to severe tissue damage, organ failure and death. Assessing the extent of organ fibrosis is crucial for accurate diagnosis of this condition. The use of Masson’s trichrome staining of tissue is a fast method for detection of morphological alterations indicative of a fibrotic phenotype. This staining method detects the extent of collagen fibers deposition and, because it employs the combination of three dyes, can also distinguish muscle fibers (red), from collagen (blue) and nuclei (black), simultaneously. After 5 min of staining 18 μm frozen sections with Harris’ hematoxylin, the sections were washed with running water. The samples were differentiated with 1% hydrochloric acid for 1 min and then rinsed for 1.5 h. Next, Ponceau (Aladdin Bio-Chem Technology, Shanghai, China) was added for 7 min, and distilled water was used for washing. The samples were treated with a 1% aqueous solution of phosphomolybdic acid (Fuguang Fine Chemical Research Institute, Tianjin, China) for approximately 5 min without washing with water and directly counterstained with an aniline blue Aladdin Bio-Chem Technology, Shanghai, China) solution for 5 min. Finally, the sample was treated with 1% glacial acetic acid (Tianjin Tianli Chemical Reagent Co., Tianjin, China) for 1 min and shaken. After staining, the slices were dehydrated with different concentrations of ethanol and cleared with 100% xylene. Cover slips were mounted over the sections before they were assessed under a microscope.

### 2.10 Immunohistochemistry

The histological sections were subjected to antigen retrieval, cooled for 2 h, treated with 0.3% (w/v) H2O2 for 15 min, blocked with PBS containing 5% (w/v) bovine serum and 0.1% (w/v) Triton X-100 for 15 min, and incubated with GFAP antibody (dilution ratio: 1:500, ab10062, Abcam) and NeuN antibody (dilution ratio: 1:200, ab177487, Abcam) overnight at 4°C. After the sections were washed, they were incubated with biotinylated secondary antibodies (1:200, Thermo Fisher Scientific, United States) for 1 h, followed by staining with the avidin–biotin–peroxidase complex (ABC, Vector Laboratories, United States). The diaminobenzidine (DAB) reaction was visualized from the immunoprecipitated product. After the aforementioned staining procedures, the sections were dehydrated with different concentrations of ethanol and cleared with 100% xylene. Cover slips were mounted over the sections, which were then assessed under a microscope (Carl Zeiss, Oberkochen, Germany). The expression levels of MPO, Zo-1, GFAP and Neun were assessed in the sections. Post-sectioning, antigen retrieval was conducted at high temperature, and non-specific binding sites were blocked with 5% bovine serum albumin (BSA). The antibody incubation process involved the use of MPO antibody (dilution ratio: 1:500, ab90811, Abcam), Zo-1 antibody (dilution ratio: 1:200, ab221547, Abcam), GFAP antibody (dilution ratio: 1:500, ab10062, Abcam) and NeuN antibody (dilution ratio: 1:200, ab177487, Abcam) overnight at 4°C. Immunohistochemical sections were observed using microscope.

### 2.11 Immunofluorescence

Following behavioral testing, mice were anesthetized with 25% urethane and transcardially perfused with 0.9% NaCl solution to flush the brain. The brains were then extracted and immersed in 4% paraformaldehyde (PFA) solution for 24 h. Subsequently, the brains were transferred to a 30% sucrose solution until they sank, retrieved, embedded in O.C.T. compound, and coronal brain sections (15 μm) of the hippocampal region were obtained using a cryostat (Leica). The brain sections were permeabilized with 0.1% Triton X-100 and then incubated with an ROS antibody (dilution: 1:500, 309800, Sigma) for 30 min at 25°C. Nuclei were counterstained with DAPI (dilution: 1:1000, ab285390, Abcam). Finally, immunofluorescence images were acquired using a fluorescence microscope.

### 2.12 Whole-cell recording

In this study, mice were adequately anesthetized with 25% urethane, and rapid brain slices were obtained. After a period of recovery, brain slices were placed in a recording chamber on a microscope, with a constant temperature perfusion system maintaining a temperature of 28.5°C in a 25°C environment. Oxygen-saturated artificial cerebrospinal fluid (ACSF) was continuously perfused at a rate of 2 ml/min. Throughout the perfusion, a non-competitive GABA receptor antagonist, picrotoxin (PTX) at a concentration of 100 μM, was added to eliminate the impact of GABAergic inhibitory synaptic activity. Glass microelectrodes (10 cm long, inner diameter 0.1 mm, outer diameter 1.2 mm) were pulled three times using a horizontal electrode puller to form a sharp-tip open electrode. After polishing with an electric microforge, approximately 0.5 cm of the electrode was filled, and any air bubbles were gently removed by tapping. The desired impedance for the electrode after solution filling was 4.5-6.5 MΩ. Data acquisition was performed using a Multiclamp 700B patch clamp amplifier, Digidata 1550A processor, and Clampex 10.5 sampling software, with a data sampling frequency of 10 kHz and low-pass filtering at 1 kHz. Current clamp mode was employed to measure neuron intrinsic excitability induced by current. Following the establishment of whole-cell recordings, a current of ±50 pA was injected to adjust the cell membrane potential to -70 mV. Neuronal discharge was then recorded under step current stimulation: a 1000 ms duration, ranging from 0 to 300 pA in 25 pA steps, with a 10 s interval. A hyperpolarizing current of 40 pA lasting 150 ms was applied before each sweep to measure neuronal voltage changes. Input impedance was calculated based on Ohm’s law, and cells with input impedance variations within a 20% range throughout the entire recording were selected for analysis. Subsequently, voltage clamp mode was switched to record I_h_ currents.

### 2.13 Microbiota analysis

Fecal samples from all groups were collected at baseline and endpoint of this study, and samples were stored at -80°C for analysis with the use of 16S rRNA-based high-throughput sequencing. Fecal samples were collected and analyzed, as previously reported. Specifically, the 16S V3-V4 regions were amplified based on the following primers: forward primer: 5’ CCTACGGGNGGCWGCAG 3’; and reverse primer: 5’ GACTACHVGGGTATCTAATCC 3’ (Abellan-Schneyder et al., 2021). Products from each sample were mixed at equal concentration, and were then analyzed by following standard Illumina sequencing protocols. According to the characteristics of the amplified 16S region, a small fragment library was constructed and dual-indexed paired end Illumina sequencing was performed on the Illumina NovaSeq sequencing platform. The Silva Release 138.1 was used as database for classification of 16S rRNA gene amplicon reads. The results of 16S rRNA were analyzed by Mothur, UPARSE, and R. Operational taxonomic units (OTUs) were clustered at 97% similarity and filtered by the UPARSE pipeline. Alpha diversity refers to the diversity within a particular environment or ecosystem and Beta diversity refers to the species differences between different environmental communities. Beta diversity and alpha diversity together constitute the overall diversity or biological heterogeneity of a given environmental community. Chao1 and Shannon were analyzed by mothur, while mainly used to reflect the richness and evenness of species. Bray-Curtis distances were analyzed by mothur, while data visualization was achieved by principal coordinate analysis (PCoA) in R. Significance thresholds were adjusted based on a false discovery rate when making multiple comparisons by the Benjamini–Hochberg approach.

### 2.14 Statistical analyses

The data are presented as the mean ± standard error of the mean (SEM), and the analysis was conducted using GraphPad Prism 9. Dealing with three or more groups, analysis of variance (ANOVA) was utilized to compare differences among them. In the case of repeated measures designs, repeated measures ANOVA was applied. Subsequently, pairwise comparisons were performed using Tukey’s multiple comparison test. The significance level for all statistical analyses was set at p < 0.05.

## 3 Results

### 3.1 ACI induced brain-gut dysfunction and inflammation

**Figure 1:**
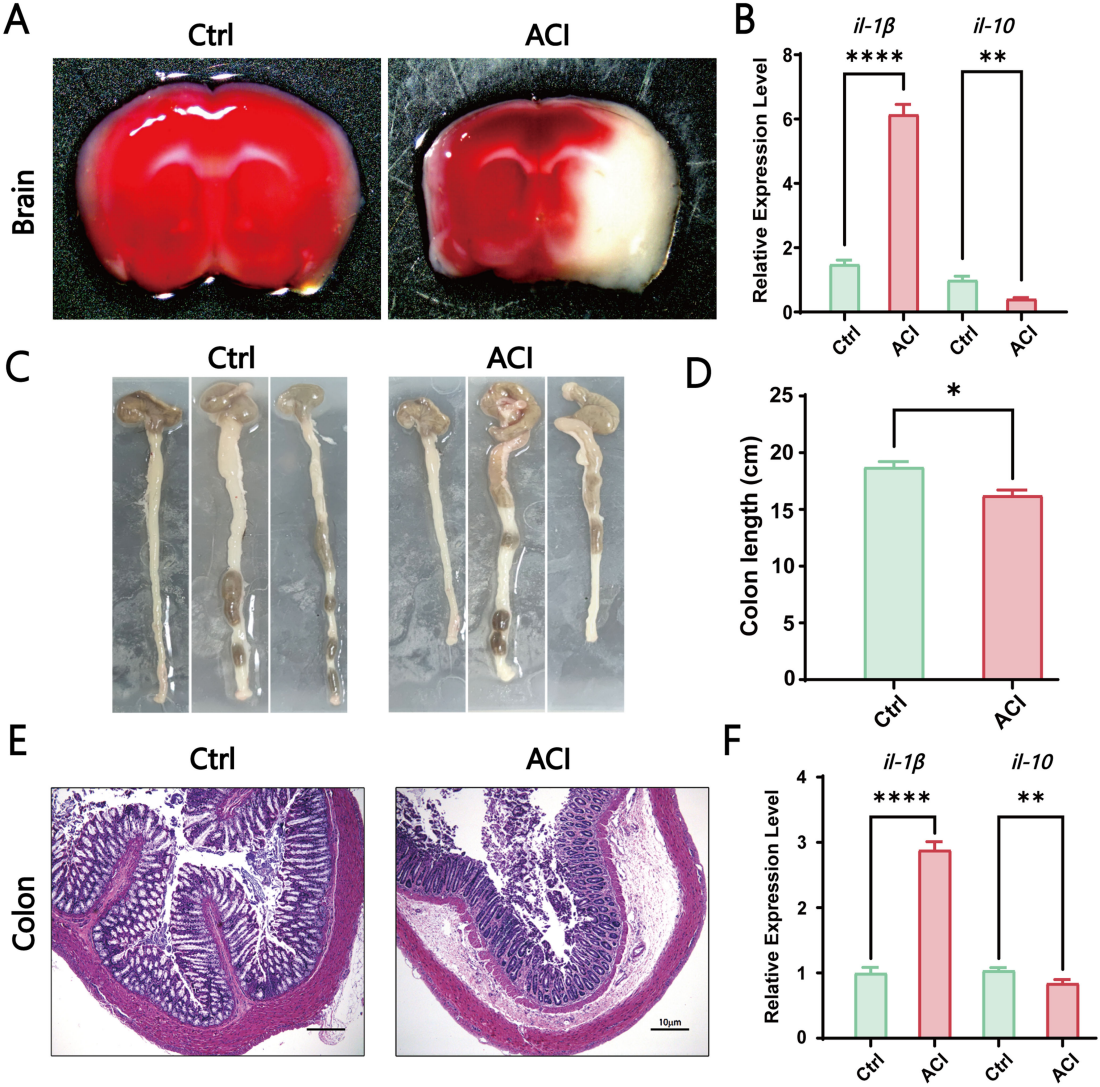
ACI-Induced Brain-Gut Dysfunction and Inflammation. **(A)** TTC staining of brains from the control group and ACI group. **(B)** Relative expression levels of *il-1β* and *il-10* in the brains of Ctrl and ACI groups (normalized to β-actin). **(C, D)** Colon lengths in the Ctrl and ACI groups. **(E)** H&E staining of colons from the Ctrl and ACI groups. **(F)** Relative expression levels of *il-1β* and *il-10* in colons of Ctrl and ACI groups (normalized to β-actin). The data are expressed as the mean ± SEM; * p < 0.05; ** p < 0.01; **** p < 0.0001, n=3.

To explore the potential role of the gut-brain axis in the progression of ACI, our study initially observed the size of the infarct area and inflammatory response in the sham surgery group (Ctrl) and acute cerebral ischemia model group (ACI). Brain infarct volume was assessed 24 h poststroke by using TTC staining. The results demonstrated a significantly larger infarct area in the ACI group than in the Ctrl group (**Figure 1A**). Concurrently, in the ACI group, the proinflammatory cytokine *il-1β* was significantly increased, whereas the anti-inflammatory cytokine *il-10* was significantly decreased compared to that in the Ctrl group (**Figure 1B**), thus indicating a pronounced inflammatory response. Given that intestinal dysfunction and inflammatory reactions are common symptoms concurrent with ACI and can reflect intestinal function, as shown in **Figures 1C, D**, ACI mice exhibited a noticeable trend of shortened colon length compared to the control group. Simultaneously, as expected, the intestines of ACI mice exhibited mild mucosal damage, thus resulting in the local aggregation of neutrophils in the mucosal submucosal layer (**Figure 1E**), along with a significant increase in the proinflammatory cytokine *il-1β* and a significant decrease in the anti-inflammatory cytokine *il-10* (**Figure 1F**). Our findings suggest the occurrence of a bidirectional immune response in the gut-brain axis during ACI.

### 3.2 The synthesis, characteristics and biosecurity of PDA

**Figure 2:**
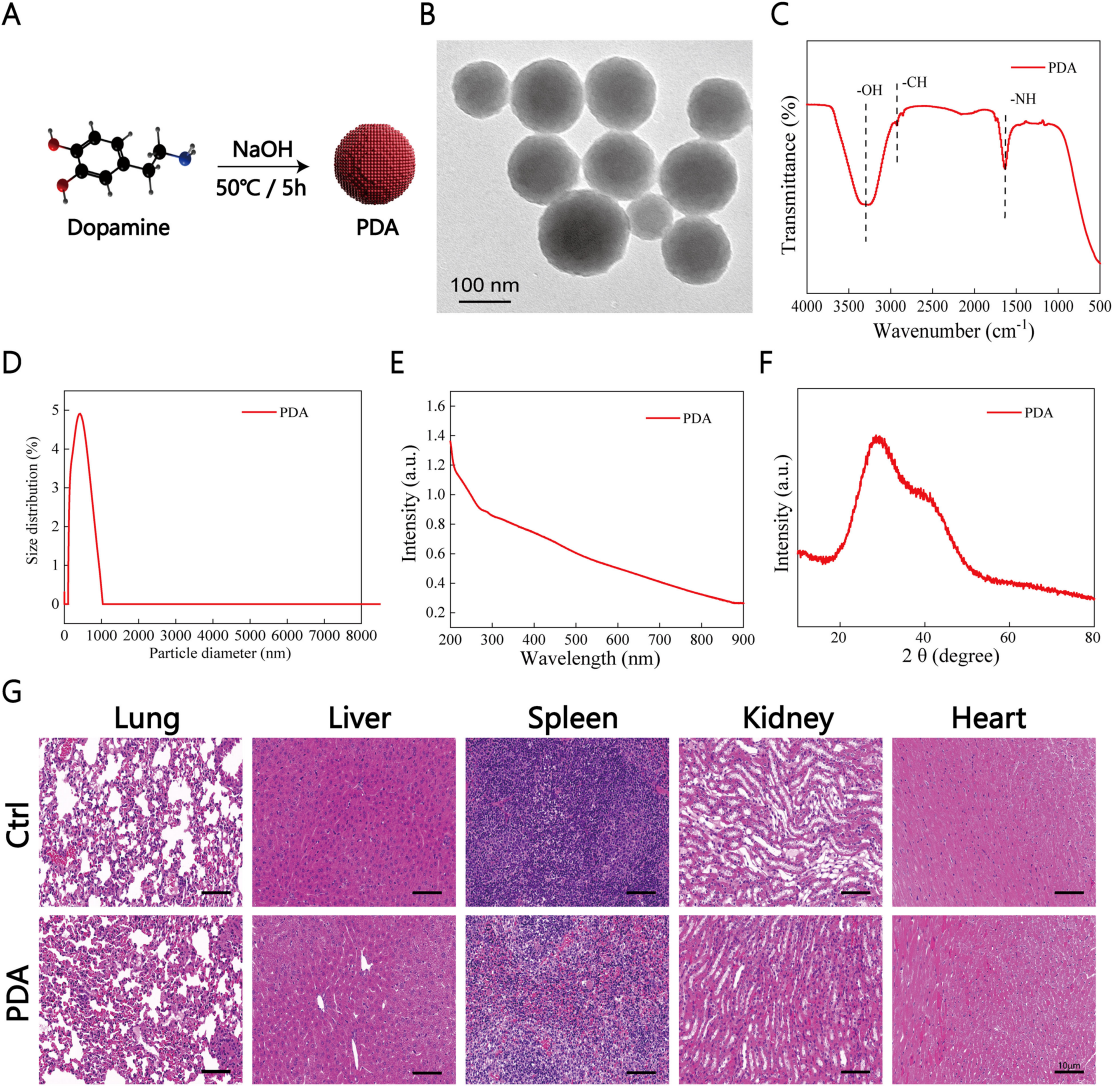
Synthesis, Characteristics, and Biosecurity of PDA. **(A)** Synthesis of PDA Particles. **(B)** Transmission electron microscopy image of PDA. **(C–F)** X-ray diffraction and infrared spectroscopy analysis of nanomaterials to validate the formation of PDA particles. **(G)** *In vivo* toxicity validation of PDA, n=3.

PDA is derived from the self-polymerization of dopamine and exhibits unique chemical properties that have garnered considerable attention in the field of nanomedicine. Despite its well-documented characteristics, the application of PDA nanoparticles as an anti-inflammatory agent for the treatment of acute inflammation-induced injuries has not been demonstrated (to the best of our knowledge). PDA nanoparticles were synthesized by using a literature-based method (**Figure 2A**) (Zhu et al., 2023).

First, the morphology of PDA nanoparticles was characterized by using transmission electron microscopy. As illustrated in **Figure 2B**, PDA exhibited a monodisperse spherical structure. Fourier transform infrared spectroscopy analysis of PDA revealed characteristic peaks at 3,300, 2,900, and 1,640 cm-1, corresponding to the functional groups -OH, -CH, and NH, respectively, in polydopamine (**Figure 2C**). The particle size distribution indicated hydrated PDA particles with a size of 424 nm (**Figure 2D**). UV–Vis spectroscopy analysis demonstrated a broad absorbance spectrum for PDA nanoparticles (**Figure 2E**). Additionally, X-ray diffraction analysis of PDA showed a diffuse halo at 2θ = 30°, thus indicating its amorphous nature (**Figure 2F**). These results collectively validated the successful synthesis of polydopamine. Subsequently, we assessed the histopathological impacts on major organs in mice treated with PDA to elucidate *in vivo* biocompatibility. Notably, no typical signals of tissue damage, such as necrosis, congestion, or hemorrhage, were observed in major organs at 14 days after a single intravenous injection of PDA (**Figure 2G**).

### 3.3 PDA relieves hemiparetic-like behaviors and local cerebral ischemia in ACI

Currently, the treatment of ACI increasingly relies on reperfusion strategies post ischemia, and the treatment window continues to expand. However, the re-establishing of blood flow in the central zone of ischemia can promote oxidative stress, amplify the release of proinflammatory cytokines, trigger a series of pathological cascades, and directly or indirectly lead to cell apoptosis, blood–brain barrier disruption, and cerebral oedema. These effects ultimately exacerbate the area of ACI. Therefore, the investigation of the role and mechanisms of postinflammatory cascades in ACI (particularly concerning PDA) is of paramount importance.

**Figure 3:**
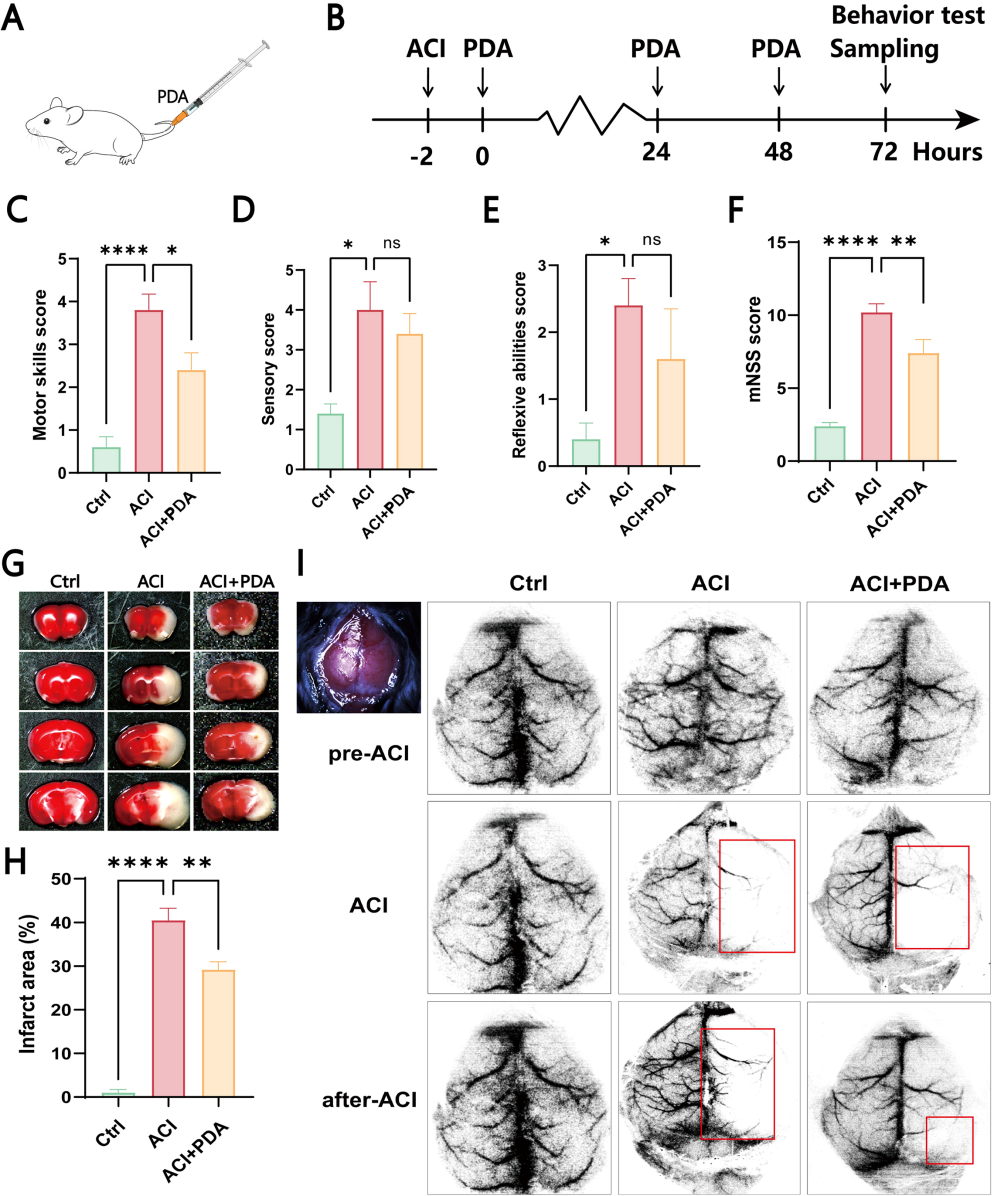
PDA Alleviates Hemiparetic-Like Behaviors and Local Cerebral Ischemia in ACI Mice. **(A)** Intravenous injection of PDA in mice tail veins. **(B)** ACI mice were injected with PDA for three consecutive days, followed by behavioral testing and sample collection. **(C–F)** Neurological function evaluation of the three mouse groups using mNSS testing on the third day post-surgery. Assessment of motor function **(C)**. Assessment of sensory function **(D)**. Evaluation of reflexes **(E)**. Total mNSS score **(F)**. **(G, H)** Results of TTC staining in the three mouse groups. **(I)** Laser speckle contrast imaging of cerebral blood flow. ns: No significant difference; The data are expressed as the mean ± SEM; * p < 0.05; ** p < 0.01; **** p < 0.0001, n=3.

First, as illustrated in the schematic diagram, we chose to intravenously inject PDA (10 mg/kg) into ACI mice at 2 h postmodelling continuously for three days (**Figures 3A, B**). Subsequently, we conducted a modified neurological severity score (mNSS) evaluation on the mice (**Figures 3C–F**). The mNSS is employed to assess the damage to mouse neurological function, and it encompasses aspects of motor function, sensation, and reflexes. In the motor test, which primarily focused on tail suspension and tabletop walking experiments to observe forelimb contraction and upright walking conditions, our results suggested that PDA alleviates the incoordination of hind limbs post-ACI (**Figure 3C**). In the sensory experiment, which mainly involved visual, tactile, proprioceptive, and balance beam tests to observe limb response delays and balance maintenance, our findings indicated that PDA alleviates the overall sensory awareness impairment in the hind limbs post-ACI (**Figure 3D**). In the reflex test, which primarily involved pinna, corneal, startle, and abnormal movement observations, as well as assessments of mouse head shaking, blinking, evasion, and seizure or spasms, our results indicated that PDA alleviates overall reflexes (including muscle spasms) post-ACI (**Figure 3E**). Finally, an overall score for mouse neurological function damage was assigned, wherein 0 indicates normal function, 1-6 indicates mild damage, 7-12 indicates moderate damage, and 13-18 indicates severe damage. As anticipated, our results demonstrated that PDA alleviates neurological function damage in mice post-ACI (**Figure 3F**).

To further confirm the neuroprotective effect of PDA on global cerebral ischemia in ACI mice, we utilized whole-brain TTC staining to assess the impact of PDA on the infarct area after ACI. The results demonstrated a significant increase in the brain tissue infarct area in the ACI group, which markedly decreased after PDA treatment (**Figure 3G**). Moreover, compared to ACI, the difference was statistically significant (**Figure 3H**), thus indicating that PDA mitigated the cerebral infarct area after ACI and exerted a neuroprotective effect. Afterwards, laser speckle contrast imaging of cerebral blood flow (CBF) was measured in all of the mice both before and after surgery (**Figure 3I**). Regions with less than 30% of baseline CBF were designated as ischemic core areas. Our findings indicated that the entire brain exhibited a high blood flow perfusion pattern before ACI. However, post-ACI, there was a significant decrease in CBF on the left side in both groups. Interestingly, this decline was markedly alleviated in the PDA group compared to the ACI group. These results confirmed that PDA treatment mitigates neurofunctional damage following ACI, thus highlighting its neuroprotective effects.

### 3.4 PDA effectively alleviates neuroinflammation and reduces astrocyte activation and neuronal apoptosis in ACI

**Figure 4:**
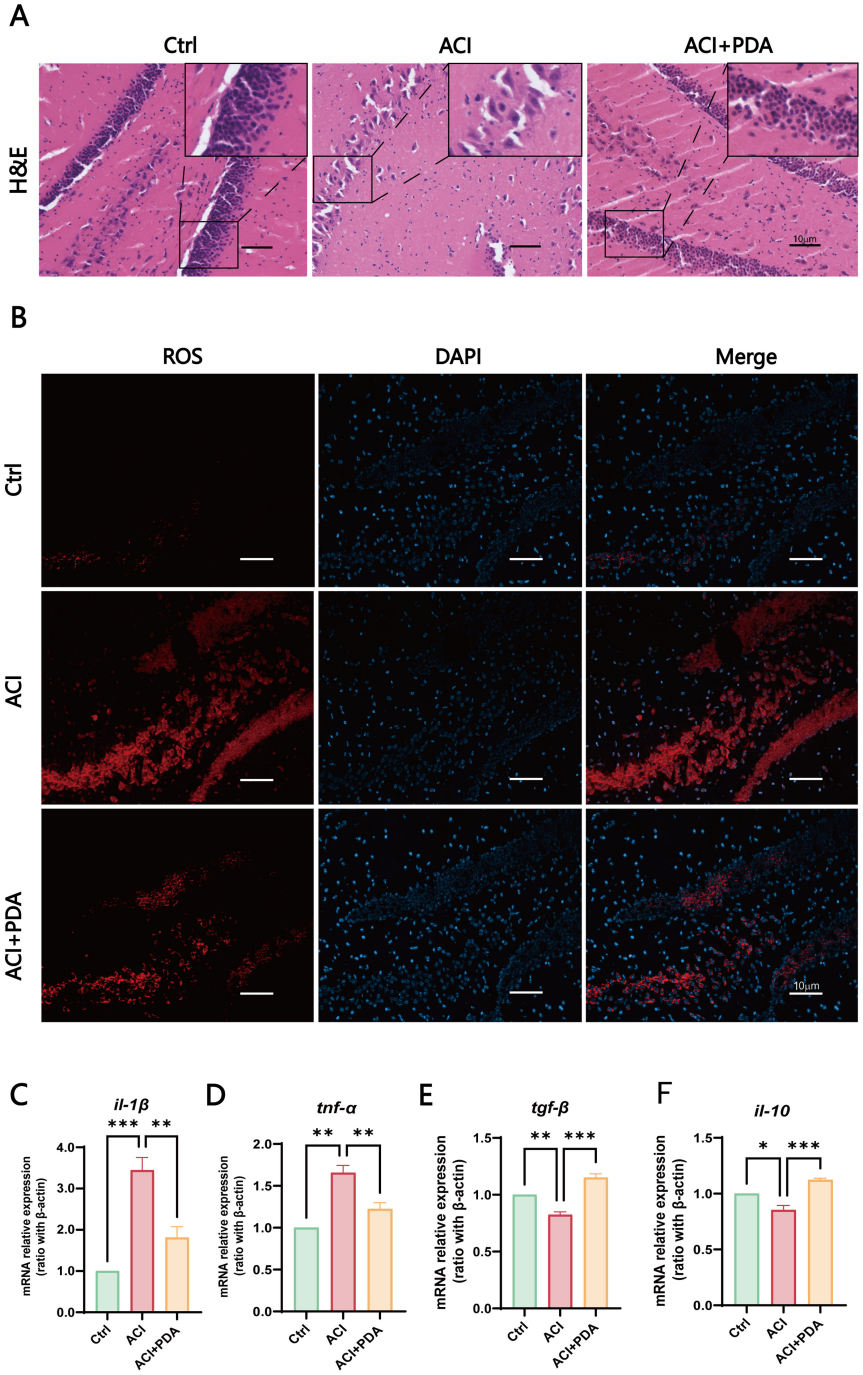
PDA Effectively Alleviates Neuroinflammation. **(A)** Assessment of pathological damage in the hippocampal region using H&E staining. **(B)** Fluorescent images of DHE staining reveal that pretreatment with PDA effectively mitigates the sharp increase in hippocampal cell ROS levels induced by ACI. **(C–F)** Expression profiles of immune cell cytokines in brain tissue determined by RT-qPCR. The data are expressed as the mean ± SEM; * p < 0.05; ** p < 0.01; *** p < 0.001, n=3.

By using H&E staining to assess morphological changes in the brain tissue following ACI, microscopic examinations demonstrated that in the Ctrl group, mouse hippocampal cells exhibited enlarged cell bodies with well-defined, intact nuclei and rich nuclear chromatin. However, in the ACI group, severe brain tissue damage was evident, which was characterized by pronounced cellular vacuolization, nuclear condensation, reduced cell count, and a network-like appearance indicative of tissue loosening. Following PDA treatment, a reduction in cellular vacuolization and nuclear condensation was observed, which was accompanied by a significant increase in cell count, thus indicating alleviation of brain tissue damage after ACI (**Figure 4A**). These findings collectively suggested that PDA improves pathological damage in brain tissue post-ACI.

Subsequently, to further assess the ability of PDA to scavenge ROS within cells, we employed dihydroethidium (DHE) as a probe. DHE can penetrate cells and, upon oxidation of intracellular ROS, generate red fluorescent ethidium (Wilhelm et al., 2009). In our experimental setup, fluorescence images of DHE-stained cells demonstrated that pretreatment with PDA effectively attenuated the significant increase in ROS levels in hippocampal cells induced by ACI (**Figure 4B**). Furthermore, we utilized RT–qPCR to examine the expression of immune cytokines in brain tissue. The results demonstrated that, compared to the ACI group, the PDA group exhibited decreased expression of the proinflammatory cytokines *il-1β* and *tnf-α* (**Figures 4C, D**). Conversely, the anti-inflammatory cytokines *tgf-β* and *il-10* showed a substantial increase in expression (**Figures 4E, F**). These differences were statistically significant, thus suggesting that PDA can effectively alleviate the progression of neuroinflammation following ACI.

**Figure 5:**
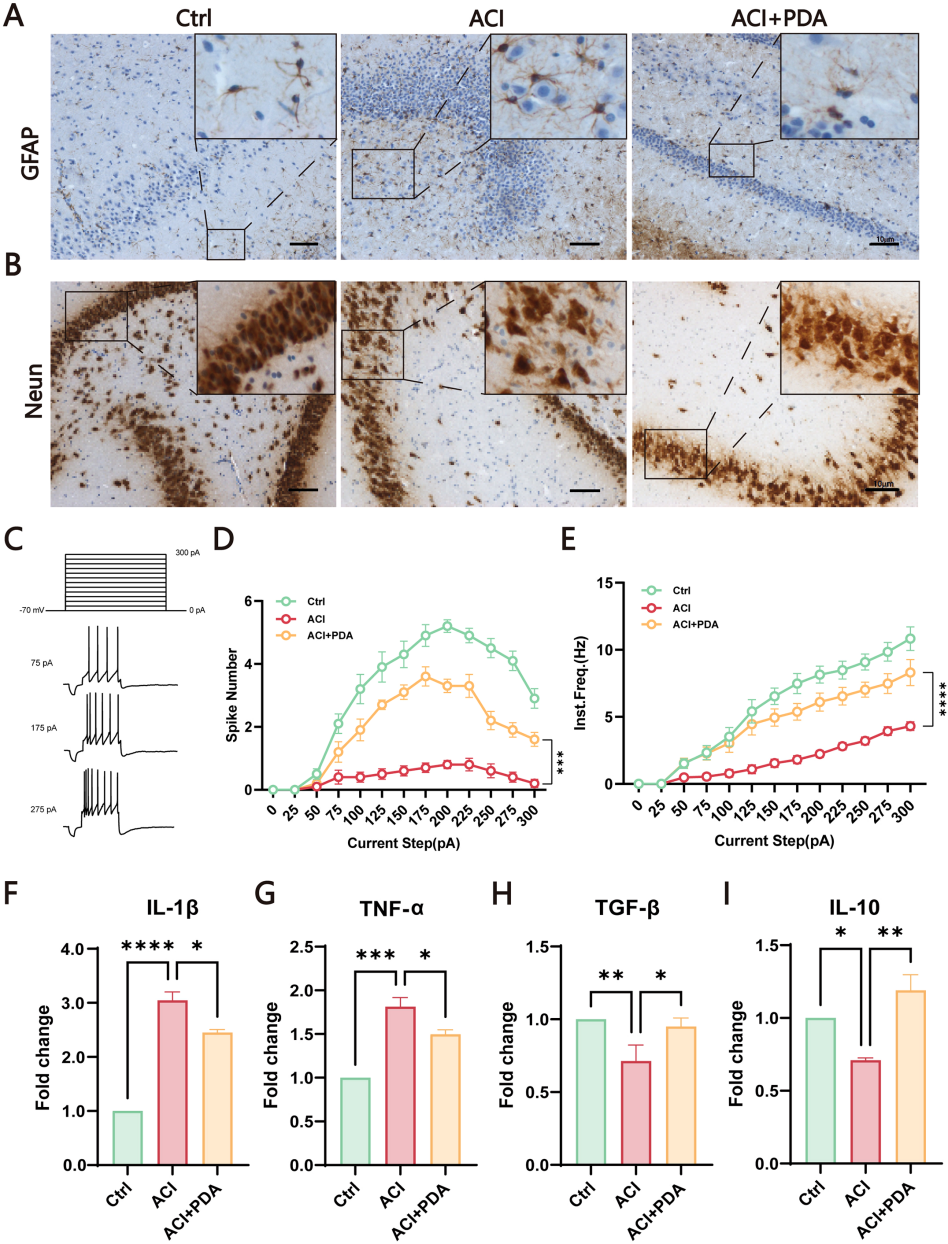
PDA Effectively Attenuates Astrocyte Activation and Neuronal Apoptosis in ACI Mice. **(A)** GFAP Immunostaining for the detection of astrocyte activation in the brain of ACI mice. **(B)** Neun staining assesses the impact of PDA on neuronal apoptosis following ACI. **(C)** Step current-induced cell action potential pattern, square wave duration 1 s, 0 pA ~ 300 pA, 25 pA step. **(D)** Number of action potentials recorded in three groups of mice. **(E)** Instantaneous frequency of action potentials in three groups of mice. **(F–I)** ELISA analysis of the expression levels of pro-inflammatory and anti-inflammatory factors in peripheral blood serum. The data are expressed as the mean ± SEM;* p < 0.05; ** p < 0.01; *** p < 0.001; **** p < 0.0001, n=3.

During ACI, neuronal damage leads to the release of excitatory amino acids such as glutamate, thus causing excitotoxic cell death. The injured neurons and astrocytes generate ROS, thus contributing to inflammation (Bao et al., 2018). To further investigate the mechanism of PDA in immune activation, we utilized glial fibrillary acidic protein (GFAP) labelled astrocytes to assess the activation status of autoimmunogenic astrocytes in the brain. The results demonstrated that, compared to the Ctrl, the ACI group exhibited an increased number of GFAP-positive cells with swollen cell bodies and reduced pseudopodial branches. In contrast, the PDA group exhibited a decrease in the number of GFAP-positive cells compared to the ACI group, with alleviated cell body swelling and increased branching (**Figure 5A**). These findings suggested that PDA can (to some extent) reduce the activation of reactive astrocytes following ACI. Subsequently, we employed neuronal nuclei antigen (NeuN) staining to examine the impact of PDA on neuronal apoptosis after ACI. The results demonstrated that apoptosis was not prominently observed in the cells of the Ctrl mice, thus indicating that the ACI surgical procedure had no significant effect on brain cell apoptosis. In the ischemic penumbra of the ACI-damaged hemisphere, a substantial increase in apoptosis was observed, thus highlighting the significant influence of ACI on neuronal apoptosis, which promoted its occurrence. Conversely, in the ischemic penumbra of the PDA group, there was a noticeable attenuation of apoptosis, thus leading to a reduction in the apoptosis rate (**Figure 5B**). This suggests that PDA can mitigate ACI-induced cellular dysregulation, thus lowering the occurrence of cell apoptosis. To gain deeper insights into neuronal activity, whole-cell recordings were utilized to evaluate the intrinsic excitability of neurons within the hippocampal region (**Figures 5C–E**). Our results indicated that the action potential discharge count and instantaneous frequency in ACI mice were significantly lower than those in Ctrl mice. Nevertheless, in comparison to the ACI group, the PDA group exhibited a significant increase in both action potential discharge count and instantaneous frequency. In summary, the administration of PDA significantly mitigated the reduced excitability levels of hippocampal neurons in ACI mice.

Given that both peripheral and central nervous system immune activation have been identified in the ACI model in previous studies, we sought to investigate whether PDA could mitigate this process. To comprehensively assess the intervention effect of PDA on immune activation, peripheral blood serum was utilized to examine the expression levels of proinflammatory and anti-inflammatory factors. The results demonstrated that, compared to the ACI group, the PDA group exhibited a reduction in the expression levels of the proinflammatory factors IL-1β and TNF-α (**Figures 5F, G**), whereas the expression levels of the anti-inflammatory factors TGF-β and IL-10 significantly increased (**Figures 5H, I**). Taken together, these findings demonstrated that the protective effects that are attributed to PDA markedly facilitated the transition to normalcy from ACI. This transition effectively led to the recovery of neuroinflammatory status, thus providing a viable platform for further exploration.

### 3.5 PDA played protective roles through the gut-brain axis and adjusted the composition and structure of gut microbes in ACI

**Figure 6:**
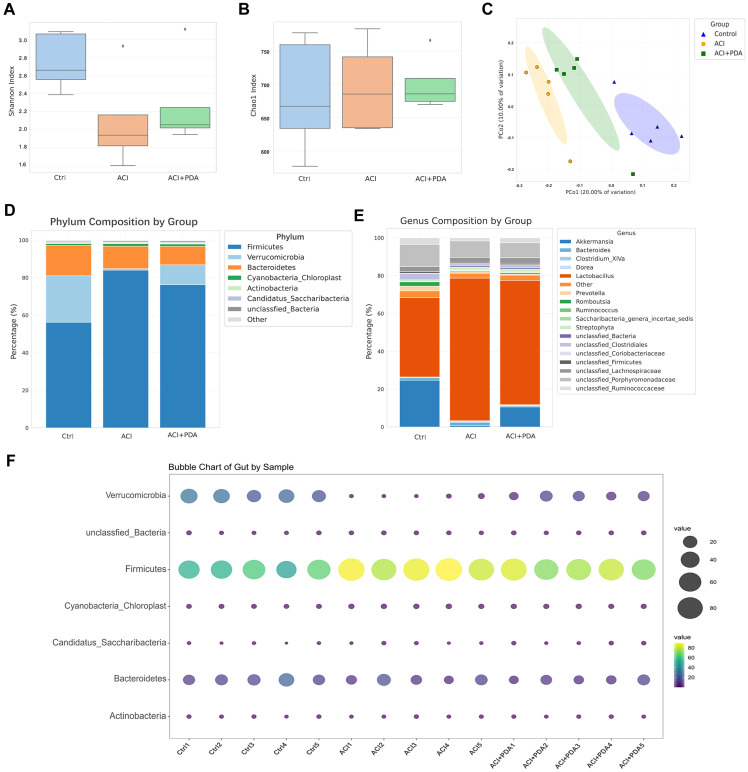
Regulation of ACI Intestinal Microbiota Composition and Structure by PDA. **(A, B)** Utilization of Shannon index and Chao1 index to assess alpha diversity. **(C)** Principal Coordinates Analysis (PCoA) based on Bray-Curtis distance. **(D)** Top 8 abundantly ranked bacterial phyla in the intestinal microbiota at the phylum classification level. **(E)** Top 18 abundantly ranked bacterial genera in the intestinal microbiota at the genus classification level. **(F)** Analysis of individual mice at the phylum classification level.

Recent research has suggested that the pathophysiological changes in ACI are mediated by bidirectional gut-brain axis connections involving the gut microbiota, intestine, and brain. Therefore, our study aimed to investigate this aspect by employing 16S rRNA gene sequencing to analyze the composition of the gut microbiota between mice treated with PDA and those subjected to simple ACI. Shannon (**Figure 6A**) and Chao1 (**Figure 6B**) indices were selected to assess alpha diversity. Meanwhile, we also analyzed the rarefaction curves for all samples and for the three groups of Shannon and Chao1 rarefaction curves for all samples and for the three groups (**Supplementary Figure S1**). The results demonstrated that, compared to the Ctrl group, the richness and diversity of the gut microbiota in the ACI group decreased, whereas in the PDA group, there was an increase in both richness and diversity compared to the ACI group. However, these differences were not statistically significant (**Figures 6A, B**). These findings suggest that ACI leads to a decrease in the species richness of the gut microbiota, and PDA treatment does not significantly alter the richness and diversity of the gut microbiota. Beta diversity reflects the overall distribution of the microbiota, and as shown in **Figure 6C**, based on Bray–Curtis distance, principal coordinate analysis (PCoA) indicated significant differences in the distribution of gut microbiota in mice after ACI compared to the Ctrl. Furthermore, the gut microbiota distribution in ACI mice tended to approach that of the Ctrl after PDA treatment. These results indicated that PDA treatment adjusted the composition and structure of the gut microbiota, thus leading to a partial convergence of the microbiota structure towards that of the Ctrl. Through the clustering operation, the sequences are divided into many groups according to their similarity, and a group is an OTU (**Supplementary Table S2**). All sequences can be OTU divided according to different similarity levels, and statistical analysis of biological information is usually carried out for OTUs under 97% similarity level. Subsequent analysis further examined the composition and structure of the gut microbiota, which generated compositional charts for the top 8 taxa at the phylum level and the top 18 taxa at the genus level for each group. At the phylum level (**Figure 6D**; **Supplementary Figure S3A**), it was found that, compared to the Ctrl group, the abundance of *Verrucomicrobia* significantly decreased in the ACI group and increased in the PDA group. *Verrucomicrobia* is known to produce short-chain fatty acids, such as propionic acid and butyric acid, which play crucial roles in regulating intestinal health and the immune system. Concurrently, in the ACI, there was a significant increase in the abundance of *Firmicutes*, which is a group of bacteria that can become opportunistic pathogens under specific conditions. At the genus level (**Figure 6E**; **Supplementary Figure S3B**), compared to the Ctrl group, the beneficial microorganism *Akkermansia*, which is known to confer health benefits to the host when present in sufficient quantities, was significantly decreased in the ACI group and increased in the PDA group. Bubbles are available to show the relationship between three variables. The horizontal axis is the sample, the vertical axis is the species classification information, and the abundance information is represented by the bubble size and color. The greener the color, the larger the bubble, and the higher the species abundance. To minimize intragroup variability, a phylum-level analysis was performed for each mouse, which yielded results consistent with those in **Figure 6D** (**Figure 6F**). These findings suggested that PDA treatment (to some extent) increased beneficial bacteria while reducing potentially pathogenic bacteria.

### 3.6 PDA effectively reversed gut dysfunction and inflammation induced by ACI

**Figure 7:**
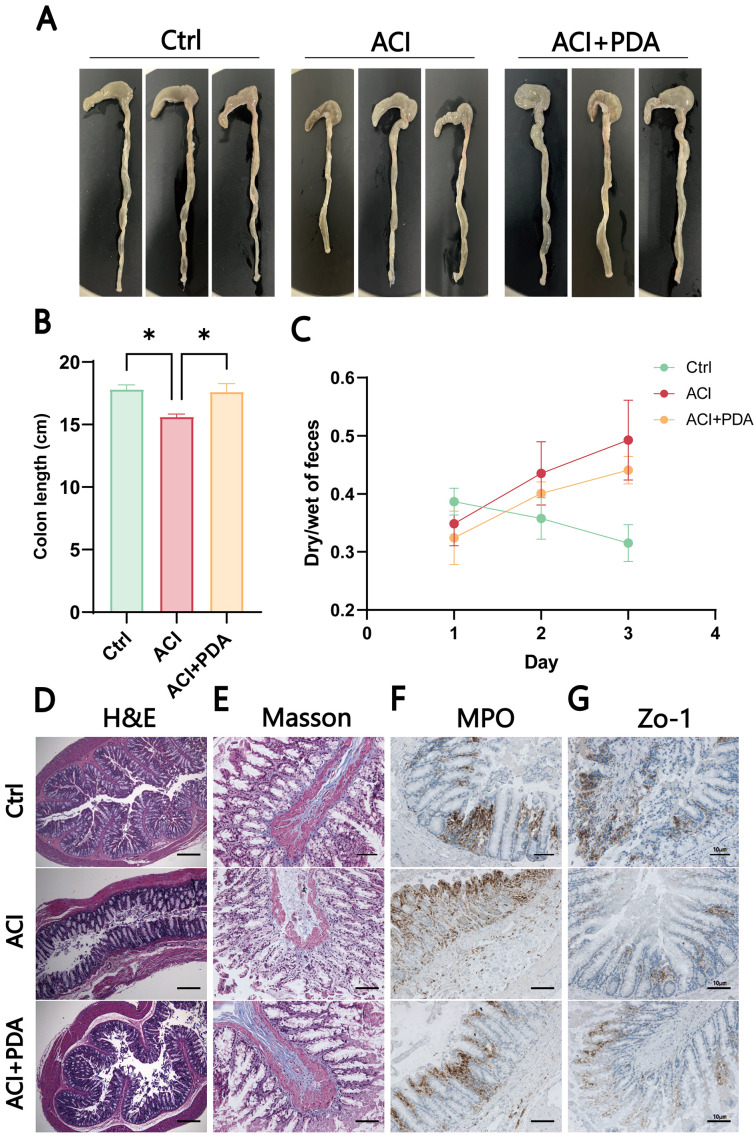
PDA-mediated induction of intestinal dysfunction and inflammation in ACI. **(A)** Photomicrographs of the colon in three mice groups. **(B)** Colon length of mice after different treatments. **(C)** Fecal wet-to-dry ratio of mice after different treatments. **(D)** Assessment of pathological damage to the intestine using H&E staining. **(E)** Evaluation of Masson area in the indicated groups. **(F)** Immunohistochemical staining to observe changes in MPO after different treatments. **(G)** IHC detection of the expression of tight junction protein Zo-1 in the colon. The data are expressed as the mean ± SEM; * p < 0.05, n=3.

After ACI, an increase in intestinal barrier permeability occurs, thus suggesting that the targeting of the intestinal barrier for protection may represent a novel approach for preventing and treating ACI. This study further investigated whether PDA affects intestinal damage following ACI. As anticipated, PDA reversed the observed colon shortening in ACI mice (**Figures 7A, B**). To assess whether PDA could ameliorate the intestinal dysfunction caused by ACI, the dry–wet ratio of feces was measured daily in each group. In comparison to the Ctrl mice, ACI mice exhibited significant constipation symptoms, which were effectively reversed by PDA treatment (**Figure 7C**). Subsequently, H&E staining was employed to analyze the histopathological changes in the small intestine tissues of each group. The results demonstrated that the small intestine tissues of Ctrl mice displayed normal villus size, slender characteristics, and an organized, tight arrangement. Moreover, ACI mice exhibited a loss of villi and a reduction in goblet cell numbers, which were partially restored with PDA treatment (**Figure 7D**). Furthermore, we explored changes in intestinal morphology, collagen fibers, and neutrophil infiltration as indicators of gastrointestinal barrier function and inflammatory status, thus providing insights into the effects of ACI and PDA. ACI mice exhibited sparse and short villi, a thinner intestinal wall, increased collagen fiber production, and elevated neutrophil numbers. PDA alleviated all of these changes, thus indicating a protective effect on the gastrointestinal barrier (**Figures 7E, F**). Based on these results, we further investigated whether PDA could protect against the loss of intestinal epithelial barrier integrity following ACI. Immunohistochemistry was employed to assess the expression of the tight junction protein Zo-1 in the colon. The results showed that the Ctrl had abundant brown–yellow Zo-1-positive expression in the intestinal epithelium. In comparison, ACI mice exhibited a reduction in the number of positive expressions, which were increased following PDA treatment (**Figure 7G**). These findings suggested that PDA treatment improves intestinal epithelial barrier damage after ACI, thus playing a positive protective role in maintaining intestinal epithelial barrier integrity.

## 4 Discussion

Neuroinflammation and excessive activation of oxidative stress constitute crucial mechanisms underlying cerebral ischemia–reperfusion injury (Yu et al., 2020; Candelario-Jalil et al., 2022). However, as of now, no antioxidant has undergone reliable clinical validation to confirm its safe and effective protection of the ischemic brain from oxidative damage. Despite prior indications from research suggesting that certain neuroprotective agents can alleviate oxidative stress and inflammatory responses in stroke patients (thereby promoting their rehabilitation in clinical practice), the hinderances posed by the blood–brain barrier and various metabolic processes during drug delivery limit the bioavailability of these drugs and escalate associated toxic side effects. Consequently, research into safe and effective antistroke drugs to enhance bioavailability and to reduce adverse effects is of paramount importance. Numerous studies have identified noninvasive nanotechnologies (such as liposomes, solid polymers, and lipid nanoparticles) as being effective means to prevent pharmacological degradation during drug transport, thus overcoming the blood–brain barrier characteristics of therapeutic drugs (Morsi et al., 2013; Sohail et al., 2023). Simultaneously, these technologies facilitate sustained drug release, thereby enhancing drug bioavailability (Chen and Li, 2020; Feng et al., 2021). Recent investigations (Shi et al., 2022) have indicated that materials based on PDA can scavenge reactive oxygen species both *in vivo* and *in vitro*, thus mitigating inflammatory responses. Nevertheless, the role of PDA-based materials in cerebral ischemia–reperfusion injury warrants further exploration. In this study, we discovered that PDA (as a therapeutic agent for treating cerebral ischemia–reperfusion injury) exhibits outstanding anti-inflammatory and antioxidant effects in the brain-gut axis interaction.

Protective Role of PDA in Neuroinflammation and Intestinal Barrier Following Acute Cerebral Ischemia. This study explored the protective effects of PDA induction against ACI, focusing on neuroinflammation and intestinal barrier preservation. Transmission electron microscopy (TEM) images of PDA samples confirmed the formation of uniform and monodisperse spherical structures, thus indicating the stable performance of our synthesized PDA nanoparticles in water without noticeable aggregation. Our data present the first direct evidence that significant immune-inflammatory responses occur in both the brain and intestines following acute ACI. Neurological deficit scores exhibited a gradual decline in mNSS scores at 24 h postbrain ischemia, thus indicating the initiation of neural recovery, which is consistent with the results of previous research. Notably, the PDA group exhibited significantly lower scores than the ACI group at 36 h, thus suggesting that PDA contributes to the recovery of impaired neural function. Ischemia–reperfusion results in the generation of a substantial amount of reactive oxygen species (ROS), which stimulates leukocyte activation and the release of various inflammatory cytokines (*tnf-α*, *il-1β*, and additional ROS). This process attacks the vascular wall, disrupts the blood–brain barrier, and leads to secondary brain injury. In this experiment, the ROS levels in the ACI group were significantly elevated compared to those in the Ctrl group, thus indicating severe oxidative stress during cerebral ischemia–reperfusion, with increased free radical activity and decreased endogenous antioxidant levels. Conversely, the PDA group exhibited improvement in these conditions. Consistent with this scenario, mice in the ACI group exhibited larger cerebral infarct areas, more severe brain oedema, and greater neurobehavioral impairments. Therefore, we propose that the therapeutic effects of PDA are closely associated with its antioxidative properties, thus enhancing endogenous antioxidant capacity and mitigating oxidative stress during ACI.

ACI promptly induces neurological dysfunction and cerebral oedema, which ultimately leads to brain death. The findings of this study demonstrated that poststroke delayed administration (PDA) can alleviate neurofunctional impairment and reduce the occurrence of ACI. The immune response and inflammation are pivotal elements in the pathobiology of ACI. The associated pathological processes are triggered within min, particularly in ischemic brain lesions and peripheral tissues, with their effects persisting for days to months or even longer. Injured and progressively dying cells in the ischemic penumbra initiate secondary inflammation, thus releasing “danger signals” that activate the immune system. Astrocytes (which are innate immune cells of the central nervous system) are rapidly activated in response to the inflammatory reaction following stroke; however, their excessive activation may exacerbate the inflammatory response. Simultaneously, astrocytes serve as crucial *in situ* regulatory factors for poststroke neuroinflammatory responses. Within 2 days to 1 week poststroke, astrocytes transition to a reactive state; in addition, by 2 weeks, scar formation occurs in the core region of the stroke. In the peri-infarct area, there is reactive proliferation of astrocytes, thus leading to the formation of glial scars, which serve to maintain central nervous system homeostasis and isolate the lesion (Bao et al., 2012). A reduction in neuroinflammation can significantly improve neurological deficits and quality of life in ACI, as well as decrease neuroinflammation mediated by activated astrocytes, thereby alleviating complications associated with ACI (Han et al., 2019; Ouyang et al., 2019; Zhang et al., 2021). Our results indicated that the addition of PDA therapy after ACI can alleviate the activation of astrocytes and suppress neuroinflammatory responses. The survival of neurons significantly influences the stability and integrity of brain function, and neuronal loss directly leads to brain functional deficits (Lazarov and Hollands, 2016). Therefore, the protection and regeneration of neurons have always been a primary focus in rescuing brain functional deficits. In ACI model mice, the number and instantaneous frequency of action potentials in hippocampal neurons were significantly lower than those in Ctrl mice. However, after PDA administration in ACI mice, the number and instantaneous frequency of action potentials remained significantly lower than those in the Ctrl group; however, compared to the ACI group, there was a significant increase in the number and instantaneous frequency of action potentials in the PDA group. These results suggested that under the influence of PDA, the excitability level of hippocampal neurons after ACI can be rescued, thus leading to a significant increase in action potential discharge frequency.

During acute ACI, there is bidirectional communication between the gut-microbiota-brain axis. ACI alters the composition of the gut microbiota. The composition of the gut microbiota in ACI/transient ischemic attack demonstrates an increase in the relative abundance of certain opportunistic pathogens and a decrease in the abundance of symbiotic or beneficial bacterial genera. This further demonstrates the detrimental impact of ACI on the composition of the gut microbiota (Yin et al., 2015). After acute ACI, up to 50% of patients may experience gastrointestinal complications, including intestinal motility disorders, dysbiosis of the gut microbiota, intestinal “leakage,” and even sepsis of intestinal origin. ACI patients who develop gastrointestinal complications typically have a relatively poor prognosis, with an increased mortality rate and severe neurological impairment. It has been reported that elderly animals may experience dysregulation of the intestinal ecosystem or intestinal-origin sepsis following ACI (Ritzel et al., 2018). Gastrointestinal complications and their potential mechanisms related to ACI are still under investigation. The experimental results indicated that, compared to the control group (Ctrl), the ACI (in terms of both microbial abundance and microbial diversity in the intestinal tract) exhibits a reduction in gut microbiota diversity. This finding is consistent with previous research reports and provides the foundation for in-depth research on this topic. An adjustment of the imbalance of the intestinal microbiota has a positive impact on the prognosis of ACI. The symbiotic bacteria in the intestines are the main source of infection for stroke patients (Kabat et al., 2014). Severe ACI leads to dysbiosis of the gut microbiota, thus resulting in reduced microbial species diversity, increased opportunistic pathogens, and consequent intestinal inflammation and immune imbalance. Mucosal-adherent bacteria, which can differ in type and are fewer in number than luminal bacteria, play a crucial role in promoting the formation of the adaptive immune system by providing low-level immune stimulation. This can induce the production of IgA, regulate the baseline expression of anti-inflammatory factors, and promote the maintenance of epithelial barrier and tight junction homeostasis. Moreover, *Akkermansia*, which adheres to the mucosa, plays a significant role in stimulating the maintenance of homeostasis in epithelial cells (Mukherjee and Hooper, 2015). After PDA therapy, there was a significant increase in *Akkermansia* in the ACI group. These findings suggested that changes in gut bacteria may be involved in the pathogenesis of ACI. Our research results also provided support (from another perspective) for the bidirectional brain-gut signaling theory.

ACI is accompanied by intestinal tissue damage, such as a decrease in intestinal barrier function. Existing research indicates that PDA can alleviate intestinal damage caused by factors such as medications, inflammation, and radiation (Zhao et al., 2019; Xiao et al., 2020). However, there is currently no research on the association and regulatory mechanisms of ACI. Intestinal epithelial cells ensure barrier integrity by selectively permeating and transporting nutrients through both transcellular and paracellular pathways (Elferink et al., 2020). This intricate connection not only provides a physical barrier against harmful molecules but also offers permeation pores for water or other solutes. The experimental results demonstrated that PDA not only induces positive morphological changes in the overall intestine of mice with ACI but also increases the distribution of neutrophils and the expression of the tight junction protein Zo-1. This suggests that after ACI, PDA has a positive effect on the integrity of the intestinal epithelium. PDA treatment can adjust the structure of the intestinal microbiota, strengthen intestinal barrier function, and reduce bacterial translocation. Simultaneously, the dysregulation of the intestinal microbiota leads to an increase in neutrophils in the intestine, thus promoting inflammatory reactions, increasing the release of proinflammatory factors, and decreasing anti-inflammatory factors, thereby enhancing neuroinflammation. This indicates that PDA may alleviate the neuroinflammatory state after ACI by affecting the migration of intestinal lymphocytes, thereby reducing brain infarction (Benakis et al., 2016). The abovementioned experimental results provide data support for elucidating the protective role of the intestinal barrier by PDA through adjusting the intestinal microbiota of ACI, increasing the integrity of the intestinal epithelial barrier, and positively regulating the immune imbalance state.

Despite the encouraging outcomes of the present study, it was not without limitations. Clearly, further research in this area is needed. Future studies should focus on neural circuits and the significance of intestinal barrier structure proteins or gut microbiota in the mechanism of ACI treatment of PDA through the use of transgenic or knock-in/knock-out species or gut microbiota intervention.

## 5 Conclusion

**Figure 8:**
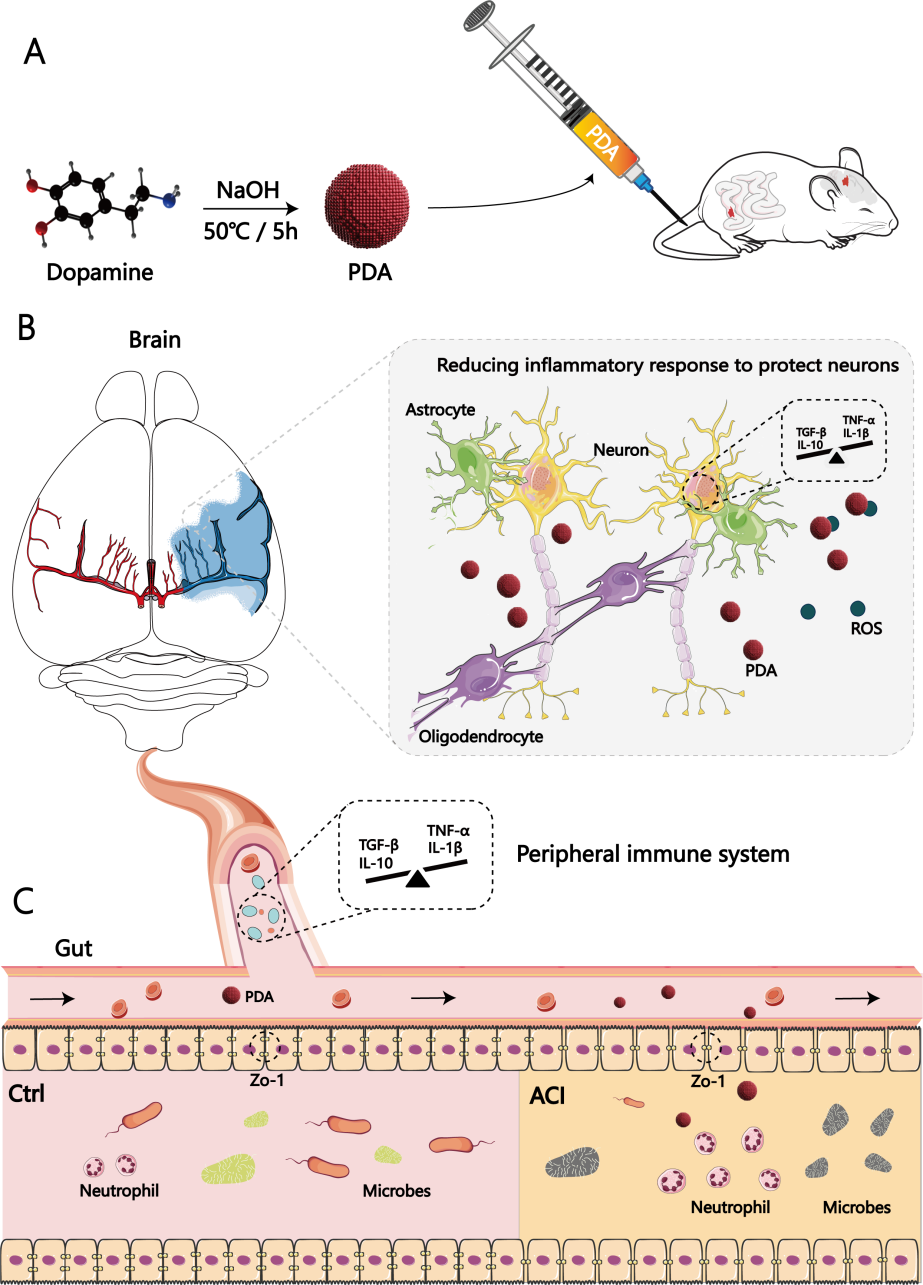
Significance of the gut tract in the therapeutic mechanisms of polydopamine for acute cerebral infarction: neuro-immune interaction through the gut-brain axis. **(A)** First, as illustrated in the schematic diagram, we chose to intravenously inject PDA into ACI mice postmodelling continuously. **(B)** PDA can reduce the generation of central ROS and the expression of pro-inflammatory cytokines after ACI. **(C)** Additionally, it alleviates peripheral intestinal dysfunction, ameliorates dysbiosis of the gut microbiota, mitigates the brain-gut immune response in ACI through its anti-inflammatory and antioxidant effects.

Via the combination of big data processing technology and data visualization, these findings provide the first evidence of ACI contribution associated with the protection of the intestinal barrier. We anticipate that our research can enhance the attention of other researchers and clinicians and promote the development of therapeutic mechanisms of PDA for ACI, specifically regarding the elucidation of the roles of gut-brain axis protection, as well as for developing more targeted clinical ACI interventions (**Figure 8**). Additionally, we also hope that gut-brain crankshaft coupling analysis should become standard practice to progress beyond a single brain or gut analysis and to acknowledge the multivariate nature of the results.

## Data Availability

The original contributions presented in the study are publicly available. This data can be found here: [https://www.ncbi.nlm.nih.gov/Traces/sra_sub/PRJNA1196102].
