# Supplementary material for: Significance of the gut tract in the therapeutic mechanisms of polydopamine for acute cerebral infarction: neuro-immune interaction through the gut-brain axis
Source: Front Cell Infect Microbiol. 2025 Mar 4;14:1413018. doi: 10.3389/fcimb.2024.1413018 (PMC11913817; doi:10.3389/fcimb.2024.1413018)
Supplement: Supplementary file 1 [file DataSheet1.pdf]

A

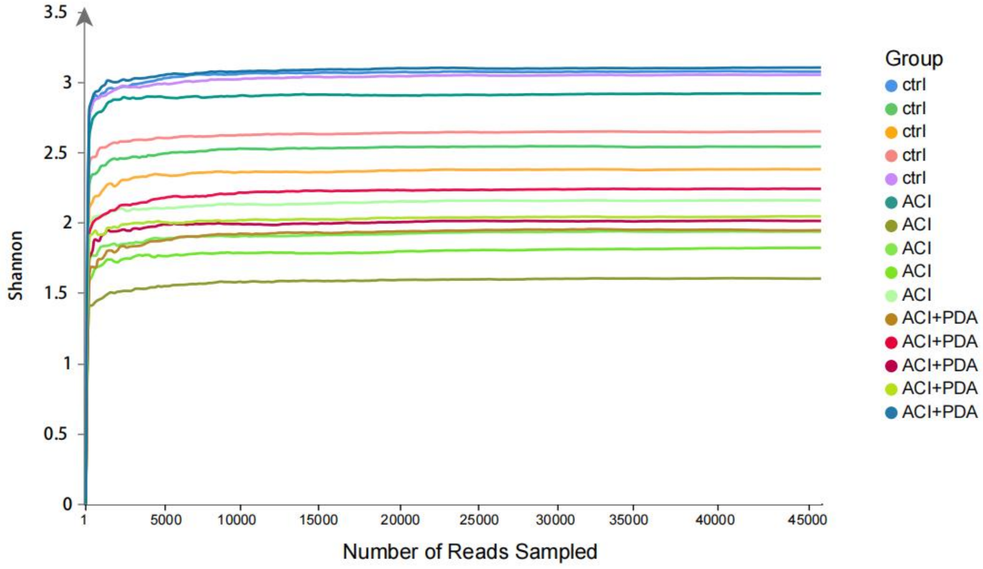
3.5
3
2.5
2
1.5
1
0.5
0
Shannon
1
5000
10000
15000
20000
25000
30000
35000
40000
45000
Number of Reads Sampled
Group
ctrl
ctrl
ctrl
ctrl
ctrl
ACI
ACI
ACI
ACI
ACI
ACI+PDA
ACI+PDA
ACI+PDA
ACI+PDA
ACI+PDA

B

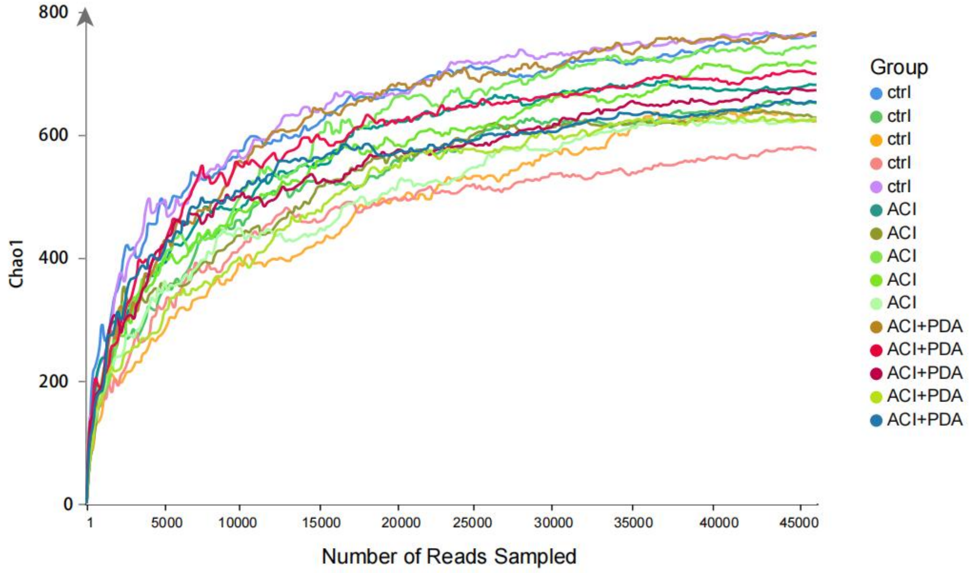
800
600
400
200
0
Chao1
1
5000
10000
15000
20000
25000
30000
35000
40000
45000
Number of Reads Sampled
Group
ctrl
ctrl
ctrl
ctrl
ctrl
ACI
ACI
ACI
ACI
ACI
ACI+PDA
ACI+PDA
ACI+PDA
ACI+PDA
ACI+PDA

C

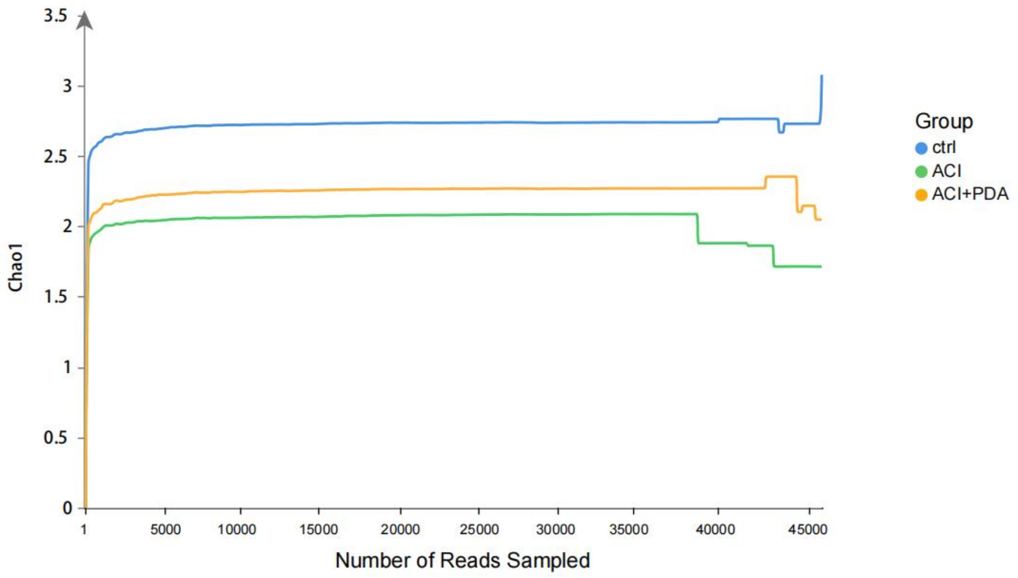

D

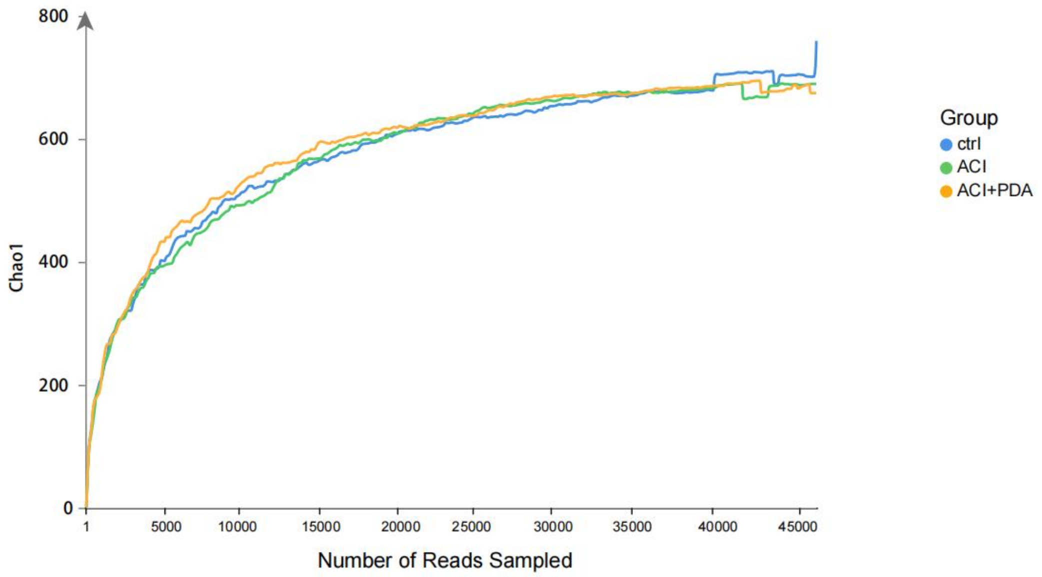

FIGURE S1. Alpha index rarefaction curves (A) The rarefaction curves for all samples of Shannon. (B) The rarefaction curves for all samples of Chao1. (C) The rarefaction curves for the three groups of Shannon. (D) The rarefaction curves for the three groups of Chao1.
