# Supplementary material for: Significance of the gut tract in the therapeutic mechanisms of polydopamine for acute cerebral infarction: neuro-immune interaction through the gut-brain axis
Source: Front Cell Infect Microbiol. 2025 Mar 4;14:1413018. doi: 10.3389/fcimb.2024.1413018 (PMC11913817; doi:10.3389/fcimb.2024.1413018)
Supplement: Supplementary file 2 [file DataSheet2.pdf]

A

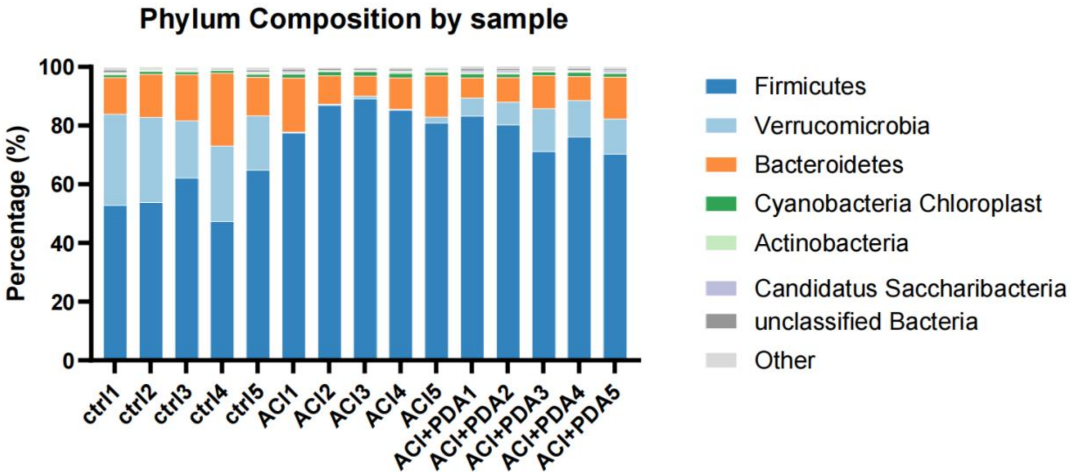

B

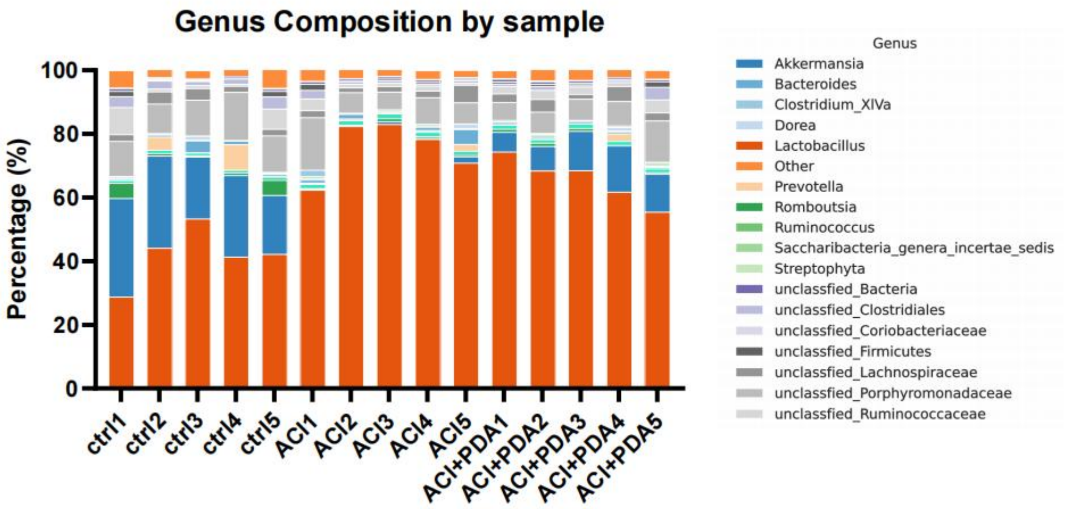

FIGURE S3. The abundantly ranked bacterial phyla in the intestinal microbiota. (A) Top 8 abundantly ranked bacterial phyla in the intestinal microbiota at the phylum classification level. (B) Top 18 abundantly ranked bacterial genera in the intestinal microbiota at the genus classification level.
